# Incidental finding of asymptomatic non-traumatic pericardial effusion in a trauma patient: a case report

**DOI:** 10.25122/jml-2022-0083

**Published:** 2023-01

**Authors:** Ahmed Alkathim, Dunya Alfaraj, Mohannad Ali Alghamdi, Samar AL-Nahash

**Affiliations:** 1 Anesthesia Department, King Saud Medical City, Riyadh, Saudi Arabia; 2 Emergency Medicine Department, King Fahad University Hospital, Imam Abdulrahman bin Faisal University, Dammam, Saudi Arabia

**Keywords:** cardiac tamponade, pericardial effusion, multiple trauma, FAST, ATLS, pericardiocentesis

## Abstract

Pericardial effusion can either be an incidental finding or a manifestation of systemic or cardiac disease. It has a wide range of presentations, from asymptomatic small effusion to rapidly progressive fatal tamponade. In a trauma setting, pericardial effusion is usually attributed to hematoma collection, with the concern of clinical evidence of tamponade that can lead to cardiopulmonary collapse. The Focused Assessment with Sonography for Trauma (FAST) is a widely used tool to diagnose pericardial effusion in trauma patients. We published this case report to emphasize that the presence of pericardial effusion alone in a trauma patient does not indicate the presence of tamponade. This case concerns a 39 years old male patient who presented to ER as a trauma case after a fall from two meters height and landing on his feet. ATLS protocol was followed, and FAST showed an incidental finding of massive pericardial fluid. The trauma team was consulted, and the patient was hemodynamically stable without clinical evidence of tamponade. Echocardiography showed mitral valve stenosis and large pericardial effusion. The close observation did not suggest the presence of cardiac tamponade. The pericardial catheter was inserted during admission with drainage of 900cc of serous fluid. The presence of pericardial fluid in a trauma setting does not confirm the diagnosis of tamponade. The mechanism of injury, clinical presentation, and the patient's stability are essential factors in determining further management of such patients.

## INTRODUCTION

In clinical practice, pericardial effusion can either be an incidental finding or a systemic or cardiac disease manifestation. Pericardial effusions range from mild asymptomatic effusions to life-threatening cardiac tamponades, with etiologies varying between infectious, neoplastic, autoimmune, metabolic, drug-related, or traumatic [1]. The scientific literature suggests that a significant number of asymptomatic pericardial effusions have been and will be identified [2–4]. The Focused Assessment with Sonography for Trauma (FAST) is an accurate and rapid tool for an initial evaluation in the trauma setting [4]. However, echocardiography is considered the imaging modality of choice to assess the pericardium [5] because it is essential to identify pathophysiologic alterations such as chamber collapse, inferior vena cava plethora, and marked respiratory variation in mitral and tricuspid inflow [5, 6].

## CASE REPORT

This case report describes the presentation of a 39-year-old Egyptian male brought to the emergency department (ED) after falling from a height of approximately two meters and landing on his feet. The patient presented one hour after the incident, fully awake and oriented, and was hemodynamically stable upon arrival. He reported experiencing pain but did not present with any signs of respiratory distress.

According to the Advanced Trauma Life Support (ATLS) protocol, the airway was assessed and found to be patent, and a C-collar was applied in the ED. He had normal breathing, equal bilateral air entry, and a muffled heart sound. The chest was equally rising with no contusions, flail chest segment, or tenderness. Oxygen saturation was maintained on room air.

During the circulation assessment, the patient was conscious and alert, maintaining normal blood pressure and heart rate with no signs of shock. A puncture trauma with minimal bleeding was found on the right leg, and two large bore IV lines were established to secure and manage circulation. Regarding disability, the patient was conscious, alert, and oriented with a Glasgow Coma Scale (GCS) of 15/15 and bilaterally reactive normal pupils. Exposure of the patient and log roll examination was performed, which revealed tenderness at the lumbar area with no deformity, step-off, or wounds. After that, he was kept dry and warm.

### Adjunct to primary survey

**Figure 1:**
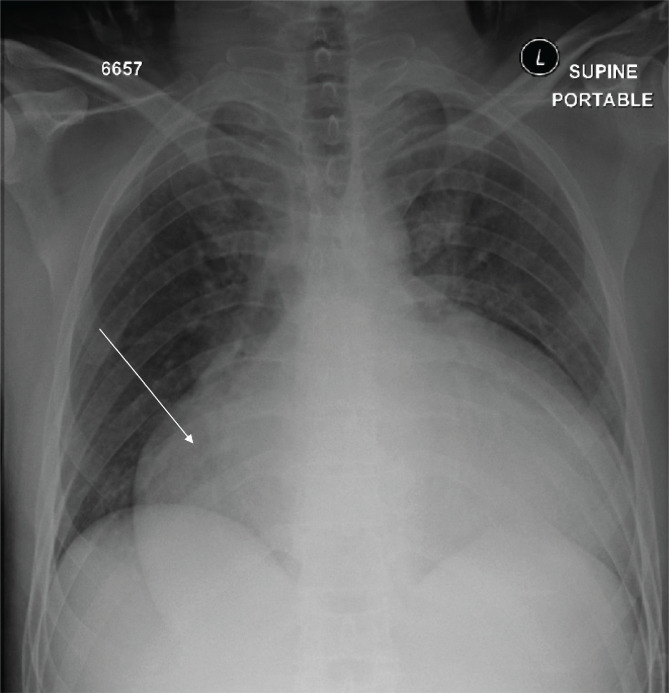
Chest X-ray; the arrow indicates the massive pericardial effusion.

Vital signs upon presentation were as follows: heart rate: 69 beats per minute, blood pressure: 127/72 mmHg, temperature: 36.9℃, respiratory rate: 19 breaths/min, and SpO_2_: 99% on room air. An electrocardiogram (ECG) was performed, and the results were normal. The chest x-ray (CXR) revealed a massive pericardial effusion, as evidenced by an enlarged cardiac silhouette and clear lungs (Figure 1). A pelvic x-ray was also performed and was found to be unremarkable. Finally, the Focused Assessment with Sonography for Trauma (FAST) revealed a massive pericardial effusion with no other significant findings.

### Secondary survey

The patient's medical history was unremarkable, except for a previous diagnosis of rheumatic heart disease and mitral valve disease. During the physical examination, no significant findings were noted except for muffled heart sounds without jugular venous distension, a puncture wound with minimal bleeding on the anterior aspect of the right leg, and redness, tenderness, and swelling at the right heel.

### Adjunct to secondary survey

#### Radiographic evaluation

**Figure 2:**
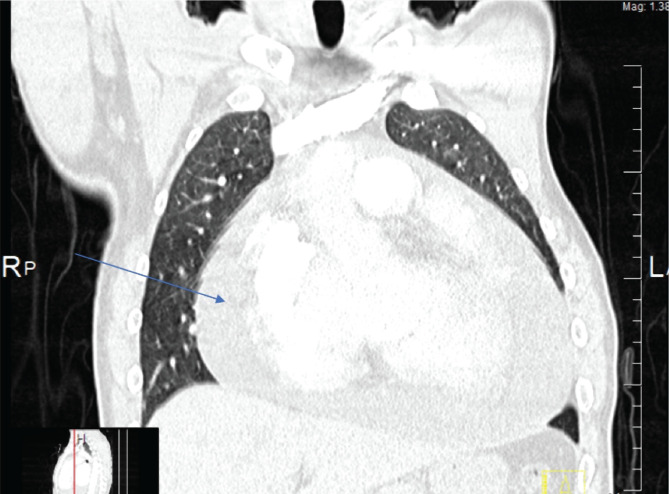
Chest CT coronal view; the arrow indicates the massive pericardial effusion.

The patient was transferred to the radiology department for a pan-CT scan, which revealed the following findings:

Chest: extensive pericardial effusion with significant left atrial enlargement and mitral valve calcification (Figure 2). No evidence of trauma-related injury was found. The CT scan was not conclusive in ruling out major vessel injury.

Spine: the scan revealed L1 and L2 wedge fractures. Otherwise unremarkable.

X-ray of the right foot: revealed a comminuted calcaneal fracture, confirmed by a foot CT.

**Figure 3:**
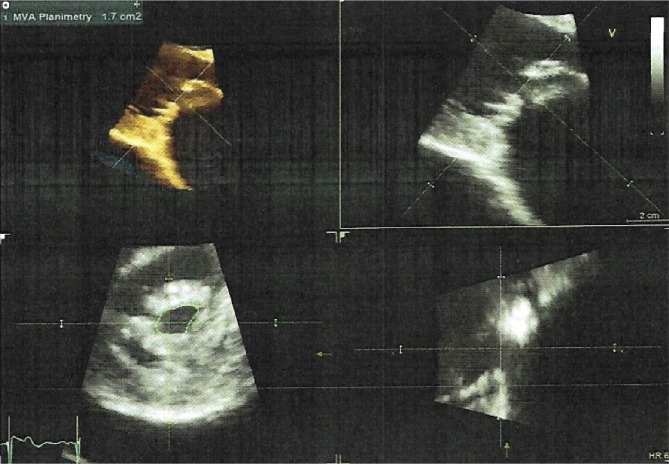
Echocardiography.

**Figure 4:**
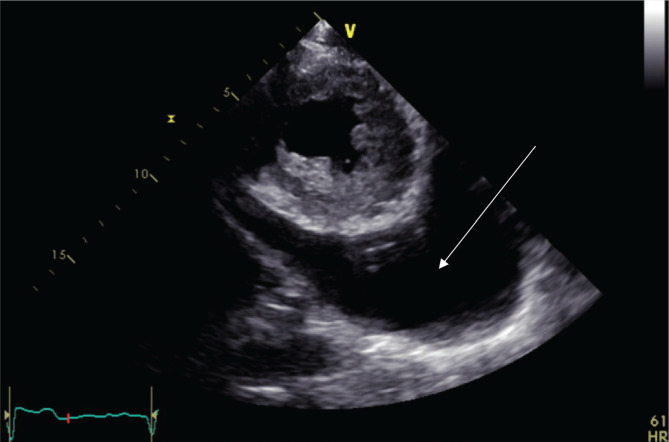
Echocardiography, short parasternal view.

**Figure 5:**
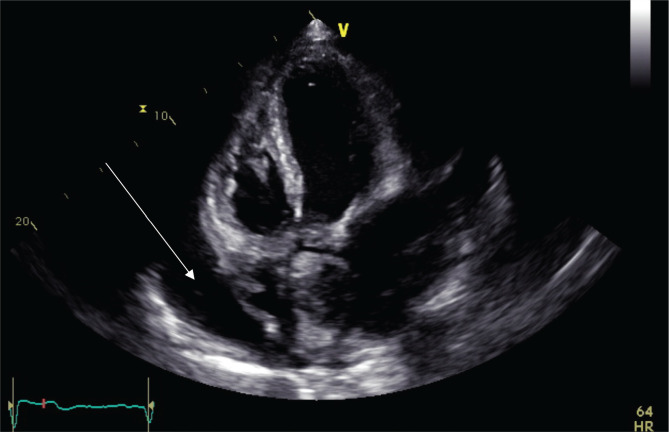
Echocardiography, apical four chambers view.

Echocardiography: showed normal systolic function with ejection fraction (EF) of 66% without regional wall motion abnormality. Normal inferior vena cava with respiratory change in dimension >50%. Large pericardial effusion with mitral valve thickening and tethering of the leaflets tips, with moderate mitral stenosis. Mitral valve area (MVA) by 3D planimetry was 1.7 cm^2^ mean gradient 6.5mmHg with mild to moderate mitral regurgitation, posteriorly directed jet. Wilkins' score was 10/16 (Figure 3). In the short parasternal view (Figure 4), a large pericardial fluid collection is shown. Finally, in the apical four-champers view (Figure 5), there was circumferential pericardial effusion with normal right ventricular and right atrial shape without diastolic collapse, making the possibility of tamponade less likely.

Laboratory tests were unremarkable, with normal renal and liver function tests, normal venous blood gas with normal base excess, normal coagulation profile, and normal cardiac enzymes. Complete blood count (CBC) was unremarkable, apart from a slightly elevated white blood count (WBC) of 17.3 K/ul (normal level 4 – 11 K/ul).

Consultation: trauma team, cardiologist, and orthopedics were involved in this case.

### Transfer to definitive care

The patient did not present any clinical signs indicating cardiac tamponade apart from muffling heart sounds. The patient was not dyspneic or tachycardic. He had a normal breathing pattern, oxygen saturation, and blood pressure. No jugular venous distension was noted. The patient reported no history of dyspnea or orthopnea prior to the trauma.

However, two hours after admission, following manipulation of the right leg by the orthopedics team while applying a cast, the patient presented with drowsiness, dyspnea, and sweating. He was found to have hypotension with a blood pressure of 80/40 mmHg. The ATLS protocol was initiated again, and it was concluded to be a vasovagal response. Blood pressure was restored by 1 liter of IV fluids, after which the patient's condition improved.

According to the ED team, trauma team, and cardiologist, this was a vasovagal attack caused by the manipulation of the right leg by the orthopedic surgery team to apply a cast. To further investigate the etiology of the massive pericardial effusion, an elective pericardiocentesis was performed by the cardiothoracic surgeon, and a pericardial catheter was inserted. Initially, 250cc of serous fluid was drained, with a total of 900cc drained until the catheter was removed a few hours later. Unfortunately, fluid analysis was not performed.

The patient was then admitted under cardiac surgery to manage the pericardial effusion and moderate mitral stenosis. The patient was scheduled for mitral valve replacement surgery, but the operation was not followed through due to the patient's wishes. The patient was later discharged against medical advice.

## DISCUSSION

James and Franklin reported four cases of patients who underwent surgeon-performed ultrasound after blunt truncal injuries and were found to have pericardial effusion. Their findings were described as "incidental" pericardial fluid. The authors mentioned that all patients were hemodynamically stable, had significant underlying conditions, sustained minimal or no injuries, and underwent surgeon-performed ultrasound of the precordial area during a routine sonographic evaluation that follows thoracoabdominal trauma [2].

Felder reported a case where a hemodynamically unstable patient, following a stab wound to the thoracoabdominal region, was found to have pericardial effusion. By emergent thoracotomy, the effusion appeared non-traumatic with no relation to the hemodynamic instability. The cause of the patient's pericardial effusion was identified as disseminated coccidioidomycosis by histopathologic analysis [7].

Initially, it can be challenging to identify the etiology of pericardial effusion in many patients as no apparent cause is present when the effusion is first identified [8]. Volk and Surg found that in 76% of cases, the use of cytology, microbiology, and pathology for fluid analysis during pericardial drainage of non-traumatic pericardial effusion was unable to identify the underlying disease process responsible for the effusion. This study found that pericardial drainage can be an effective therapeutic tool, but it is a limited diagnostic modality when it comes to identifying the cause of the effusion [9]. However, the study also highlighted that clinical findings such as the presence or absence of underlying conditions, as well as the presence or absence of inflammatory signs like chest pain, fever, and friction rub, can help classify patients into a major etiologic diagnostic category. This is supported by Sagristà-Sauleda and colleagues in a retrospective study of 322 patients with underlying conditions that could lead to effusion. In the study, the underlying condition was, in fact, the cause of the presenting pericardial effusion in all but 7 patients [8]. Thus, by considering clinical history, physical examination, laboratory investigations, and appropriate imaging, a reliable conclusion can be reached regarding the etiology of pericardial effusion. Table 1 compares the characteristics of patients in the reported cases to our patient.

**Table 1:** "Incidental" pericardial effusion characteristics in literature.

Author	No. patients	Gender	Age (years)	Mechanism of injury	Hemodynamics	Type of fluid (if drainage was done)	Final diagnosis
**Our case**	1	M	39	Fall	Stable	Serous	Rheumatic mitral stenosis (Confirmed by Echocardiography)
**Lukan JK & Franklin [2]**	1	F	82	Motor vehicle collision	Stable	Non-bloody effusion	Not mentioned
	2	F	77	Motor vehicle collision	Stable	Non-bloody effusion	Not mentioned
	3	F	75	Motor vehicle collision	Stable	Not done	Not mentioned
	4	M	90	Motor vehicle collision	Stable	Not done	Not mentioned
**Felder SI [7]**	1	M	50	Stab wound	Unstable	Sero-purulent	Disseminated coccidioidomycosis (Confirmed by histopathology)

The current case is an example of an incidental finding of an asymptomatic massive pericardial effusion in a trauma case without a history of blunt or penetrating thoracic trauma, suggesting a non-trauma-related effusion. This is supported by several factors: (1) the patient had a history of rheumatic heart disease, which has been linked to pericardial effusion by multiple studies [1, 6], (2) the presence of mitral stenosis, which has been recognized as a cause of pericardial effusion [1, 6], (3) the pericardial fluid aspiration showed a serous collection, and (4) the lack of clinical symptoms or signs of cardiac tamponade supports the conclusion that it is not a case of cardiac tamponade. Additionally, the mechanism of trauma and the absence of trauma-related thoracic injuries point to a low possibility of pericardial effusion secondary to trauma.

## CONCLUSION

The presence of pericardial fluid in a trauma setting does not confirm the diagnosis of tamponade. The mechanism of injury, clinical presentation, and the patient's stability are important factors in determining the further management of such patients. This case report serves as an audit that might indicate a pattern in trauma cases presenting to the emergency department.

## References

[1] Imazio M, Adler Y (2012). Management of pericardial effusion. European Heart Journal.

[2] Lukan JK, Franklin GA, Spain DA, Carrillo EH (2001). "Incidental" pericardial effusion during surgeon-performed ultrasonography in patients with blunt torso trauma. J Trauma.

[3] Nagy KK, Lohmann C, Kim DO, Barrett J (1995). Role of echocardiography in the diagnosis of occult penetrating cardiac injury. J Trauma.

[4] Rozycki GS, Feliciano DV, Ochsner MG, Knudson MM (1999). The role of ultrasound in patients with possible penetrating cardiac wounds: a prospective multicenter study. J Trauma.

[5] Pérez-Casares A, Cesar S, Brunet-Garcia L, Sanchez-de-Toledo J (2017). Echocardiographic Evaluation of Pericardial Effusion and Cardiac Tamponade. Front Pediatr.

[6] Vakamudi S, Ho N, Cremer PC (2017). Pericardial Effusions: Causes, Diagnosis, and Management. Prog Cardiovasc Dis.

[7] Felder SI (2015). Trauma sternotomy for presumed haemopericardium with incidental coccidioidal pericarditis. Trauma Case Rep.

[8] Sagristà-Sauleda J, Mercé AS, Soler-Soler J (2011). Diagnosis and management of pericardial effusion. World J Cardiol.

[9] Volk L, Lee LY, Lemaire A (2019). Surgical pericardial drainage procedures have a limited diagnostic sensitivity. J Card Surg.

